# Radiomics to understand pre-treatment tumor biology for resectable non-small cell lung cancer

**DOI:** 10.3389/fonc.2026.1756676

**Published:** 2026-06-02

**Authors:** Benjamin Zollinger, Duy Pham, Kei Suzuki

**Affiliations:** 1 Division of Thoracic Surgery, Inova Schar Cancer Institute, Fairfax, VA, United States; 2 University of Virginia School of Medicine, Charlottesville, VA, United States

**Keywords:** computer-aided nodule assessment and risk yield (CANARY), lung adenocarcinoma, machine learning, non-small cell lung cancer (NSCLC), radiomics

## Abstract

Lung cancer is the leading cause of cancer related mortality in the United States. The standard of care for early-stage non-small cell lung cancer (NSCLC) is surgical resection. However, with the increasing usage of sublobar resections for small stage IA tumors and neoadjuvant chemoimmunotherapy for resectable stage IB-IIIA tumors, the selection of appropriate patients and the prediction of how they might respond to such therapies is vital. Radiomics is the usage of extracted imaging features as analyzable data. Radiomic modeling with machine learning and artificial intelligence can be used to provide non-invasive and predictive information about tumor biology and aggressiveness before treatment is initiated. Radiomic modeling has been utilized to identify NSCLC histopathological features, proteomic mutational burden, responsiveness to surgical and immunotherapy interventions, and occult lymph node metastasis. This review article provides an overview of studies using radiomic features to model risk and radiomic predictive tools such as the Computer-Aided Nodule Assessment and Risk Yield (CANARY) that have been developed to provide insight and pre-operative risk stratification for resectable NSCLC.

## 1 Introduction

Lung cancer remains the number one cause of cancer-related mortality in the United States, largely due to its aggressive nature and frequent advanced stage at presentation (1–3). To counter this and promote discovery of cancers at an earlier stage, significant efforts have been made over the past two decades to increase both the efficacy and the prevalence of lung cancer screening. Published in 2011, the National Lung Screening Trial showed relative reduction in both lung cancer-specific mortality and overall any-cause mortality with annual low-dose computed topography (CT) scans when compared with annual chest X-ray in high-risk patients (4). Secondary to this landmark trial, the United States Preventative Services Task Force first published guidelines in 2013, now since revised in 2021, which currently recommend annual screening with a low-dose CT scan in adults aged 50–80 years who currently smoke or have quit within the past 15 years and have a 20 or more pack-year history (5, 6). Subsequent to these new guidelines, a large influx of screening CT imaging was generated. Between the screening CT images and other imaging necessary for workup of lung cancer, a variety of images have become available for analysis. Other lung cancer-related imaging include chest CT scans with incidentally discovered lung nodules, staging CT and positron emission tomography (PET) scans, and surveillance chest CT scans. With such a wealth of images related to lung cancer, researchers have sought to put these images and their composition to use to discern predictive and analyzable information about the underlying tumor behavior and biology. These efforts to extract and analyze quantitative information about the tumor has quickly led to the integration of the field of radiomics with the diagnosis and treatment of lung cancer.

### 1.1 Radiomics

Radiomics is defined as the extraction of quantitative features, information, and patterns from diagnostic images to use as analyzable data (7, 8). These imaging features that are extracted can include shape or size of the region of interest, particular intensity of the pixels or voxels, smoothness of the borders, relative direction, gradient, texture and distribution of the intensity of gray-level of the pixels relative to its neighbors, and much more. Once a region of interest is defined, radiomic processing software can in turn extract thousands of these informative features from the regions of interest and turn them into data points. After statistical analysis, some of these features may be used as potential biomarkers if particular patterns are associated with clinical outcomes. However, in order to develop a usable radiomics-based model for gaining inferences from the imaging, a particular workflow is followed.

After image acquisition, the area of interest for analysis must be defined and delineated on each image slice, which is a process called segmentation. This can be done manually or with semi-automated assistance, if segmentation software for the region of interest is available. Once the images are segmented, they then undergo a process of harmonization which consists of processes such as isotropic voxel spacing and outlier voxel filtering to improve consistency across images and reduce bias from acquisition factors. Radiomic feature extraction is then performed. The extraction software, which can be either institutionally developed and proprietary or publicly available, analyzes the voxel characteristics, spatial relationships, and patterns. These features are according to established standardized lists and are quantified by pre-determined algorithms and extracted. Hundreds of features may be extracted, but only a fraction of these may be relevant or contribute to a radiomic signature and used in a statistical model. These features are then selected by a process of dimensionality reduction in which redundant features are identified and eliminated by different algorithms to narrow the useable feature set and avoid overfitting. The resulting data from the selected features, can then be entered into statistical modeling pathways to draw inferences. For further overviews of the radiomics workflow, see reviews by Mayerhoefer et al., McCague et al., and Avery et al. (7–9).

For statistical analysis and inference, a multitude of different modeling algorithms can be applied. Generally, models are constructed and trained using machine learning based algorithms. Within the broader category of machine learning, these predictive models can be developed from a variety of complex algorithms including logistic regression, support vector machine, random forest, and others (10–14). The extracted features are compared across many patients within a training set of images and graded as predictors against the outcome of interest using the modeling algorithms. The generated predictive model is then tested against an external validation data set to ensure applicability and prevent overfitting to the training images. The intricacies of the development, underlying statistical formulation, and differentiation of the various machine learning algorithms are beyond the scope of this review, as are the technical details of the application of the various software packages for image processing and feature extraction. Even so, a basic understanding of radiomic methodology is necessary to understand its potential for its integration in a predictive capacity for the diagnosis and treatment of lung cancer.

### 1.2 Clinical need for pre-treatment marker of tumor biology

In recent years, treatment patterns for non-small cell lung cancer (NSCLC) have dramatically changed. New therapies for systemic treatment have been introduced, and our understanding for the extent of resection for oncologically acceptable surgical treatment has evolved. Immunotherapy with immune checkpoint inhibitors (ICI) has become an integral part of treatment plans for non-resectable and resectable disease in both the neo-adjuvant and adjuvant setting (15–21). There is currently no great predictive marker of response to the ICI regimen in NSCLC. Such marker would help clinicians in deciding appropriate treatment regimen for resectable NSCLC.

Beyond identifying biomarkers for predicting responsiveness to systemic therapies, new biomarkers for predicting the optimal surgical resection are also needed. The surgical landscape for NSCLC is changing. For peripheral stage I NSCLC ≤ 2 cm in size, the CALGB 140503 and JCOG 0802 trials showed that sublobar resection is oncologically non-inferior to the previous standard-of-care resection of a lobectomy across randomized patient populations (22, 23). These trials have subsequently raised the question of whether there might be sub-groups of peripheral stage I NSCLC ≤ 2 cm patients for which an anatomic resection, whether in the form of segmentectomy or a traditional lobectomy, might still be of benefit due to underlying tumor aggressiveness. Furthermore, approximately 10-15% of patients will have occult lymph node metastasis pathologically discovered after resection that was not apparent on pre-operative imaging. Anatomic resection will yield a higher lymph node harvest and might provide more accurate pathologic staging. At this time though, for resectable disease, the main current preoperative guiding predictor for the necessary extent of the resection is the lesion size. This fails to provide any deeper insight into the actual tumor biology or pathological features.

Therefore, it is vital that further tools are developed to offer better understanding of the underlying tumor biology beyond the standard tumor/node/metastasis (TNM) staging pre-operatively. Discernment of tumor features that might hint at an aggressive biology would better inform clinicians when deciding upon a treatment plan. With the range of oncologically adequate surgical procedures expanding in the wake of the aforementioned trials, surgeons need new tools to help decide the optimal surgery for the individual patient with resectable cancer. As radiomics offer a non-invasive approach to evaluate tumor features in the pre-treatment phase, the potential for using these models to provide nuanced information has become readily apparent. Several radiomic models have been developed with the goal of predicting cancer behavior and resulting outcomes based on preoperative imaging. This review article seeks to provide an overview of the current use and opportunities to integrate radiomic models in the pre-treatment phase of resectable lung cancer. We will discuss the use of radiomic models in the prediction of underlying aggressive tumor features once a diagnosis is made, the integration of radiogenomics and proteomics, and then for the prediction of responsiveness to treatment.

## 2 Methods

This study was conducted as a narrative review of the current published literature on radiomics and lung cancer. Our literature search was conducted using the PubMed database. Original clinical studies and meta-analyses were eligible for inclusion in the review of pertinent studies. Included studies were limited to those published in the English language, and dates of publication of included articles were limited from 2010 to the present. Basic scientific and translational studies, narrative reviews, scoping or systematic reviews without meta-analysis, single case reports, case series, published abstracts, and unpublished materials were not included. The literature search was conducted from September – November 2025. Searches were conducted using a combination of keywords and MeSH terms. Keywords and MeSH terms included were “Radiomics”, “Lung Neoplasms/therapy”, “Lung Neoplasms/surgery”, “Lung Neoplasms/diagnostic imaging”, “Lung Neoplasms/genetics”, “Computer-aided nodule assessment and risk yield”, “CANARY”, “PyRadiomics”, “deep learning”, “lung cancer”, and “resection”. Some referenced manuscripts within articles discovered from the initial searches were evaluated and included as deemed appropriate, and some previously known studies were included. Other studies providing general background information for the introduction and discussion or to provide contexts for the review subsections were cited as needed.

Strict inclusion and exclusion criteria for studies were followed. Studies that investigated the use of radiomics for lung cancer screening or the prediction of cancer risk from undifferentiated nodules were not included. Radiomic-based models that did not use validation test cohorts during development in order to prevent over-fitting of the model were excluded. Studies that used a validation cohort of n < 40 were excluded as these similarly are prone to over-fitting of models. The imaging modalities of interest were CT and PET/CT. Studies using imaging modalities that are not standard for typical pre-operative staging work-up such as magnetic resonance imaging (MRI) or radiographs were also excluded. Studies using radiomic features that were non-tumoral or peri-tumoral targeted were excluded. The scope of this review was limited to potential uses of radiomic modeling in the pre-operative setting for resectable NSCLC. Studies using radiomics to predict prognosis or response to treatment for non-resectable disease (i.e. those receiving solely systemic therapy and/or radiotherapy) were not included.

**Figure 1:**
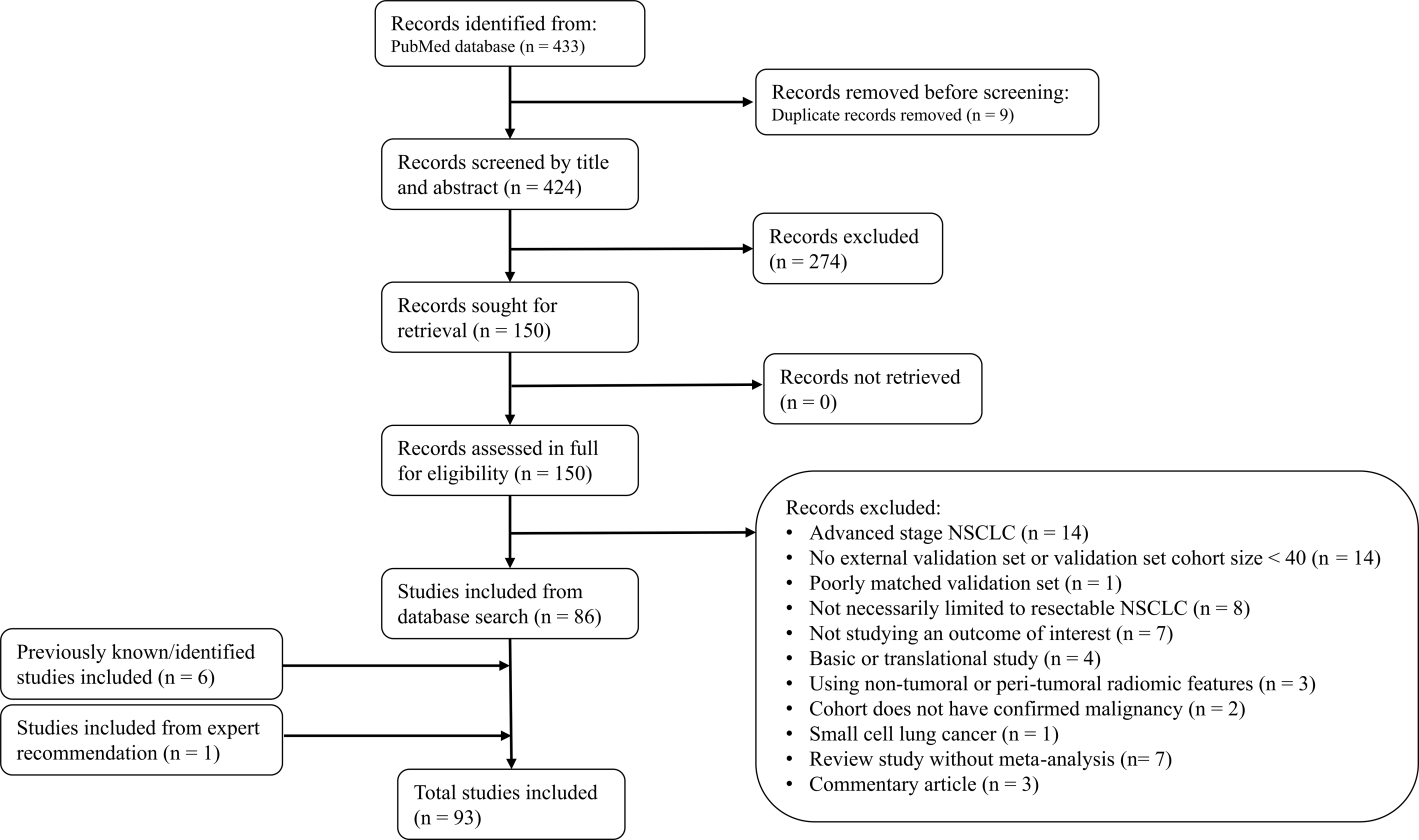
PRISMA style flowchart for study selection and inclusion for the review.

We identified 433 records on initial PubMed search. Of these, 424 studies were then screened by title and abstract by the author B.Z. after removal of duplicates. After screening, 150 records were then reviewed in full by B.Z. After exclusion of records not meeting criteria (**Figure 1**), a total of 93 studies were included. A PRISMA-style flowchart of study selection and inclusion is provided in **Figure 1** (24). This particular article is a narrative review, not a systematic review. Therefore, the studies were not directly compared to one another with heterogeneity calculated and study bias corrected for. However, using the strict inclusion criteria, we hope to maintain some relative homogeneity between study structure and minimize bias.

### 2.1 Radiomics and tumor histology

The ability to predict tumor biology and histopathology is one of the areas where radiomics has the potential to influence treatment course. Within the broader category of NSCLC, it has been shown that histologic type such as adenocarcinoma (ADC) versus squamous cell carcinoma (SCC) has profound implications for oncologic outcomes and recurrence-free survival (RFS) and overall survival (OS), even controlling for stage and with propensity score matching (25–28). Even within lung ADC, certain morphologic subtypes indicate more indolent versus aggressive tumors, such as adenocarcinoma-*in-situ* (AIS)/minimally-invasive adenocarcinoma (MIA) vs. lepidic/acinar/papillary vs. solid/micropapillary adenocarcinomas (29–32). Aggressive morphology (solid/micropapillary) have a 5-year RFS approaching stage II patients (33–37) while AIS/MIA have excellent outcome with resection alone. Knowing the histology/morphology in stage I NSCLC patients may hence play a role in the surgeon choosing the extent of resection and lymph node dissection.

#### 2.1.1 Histopathological subtypes

Radiomic models have been successful thus far in recognizing histopathological subtypes based on pre-operative imaging. These models incorporate both CT and PET imaging. Panchawagh et al. compared different machine learning algorithms to differentiate NSCLC subtypes of ADC, SCC, large cell, and undifferentiated from a cohort of 422 confirmed NSCLC cases, and they found an area under the curve (AUC) of the receiver operator characteristic (ROC) curve for detecting the correct subtype of 0.96 using random forest plot model and 0.95 for deep neural networking (38). Zhu et al. created a five radiomic feature signature to discriminate ADC from SCC in a sample of 129 NSCLC patients which was found to have an AUC of 0.89 in the validation cohort (39). Zhang et al. used a combined model of radiomic modeling using 18F-fluorodeoxyglucose (^18^F-FDG) PET/CT imaging with clinical risk factors of gender, age, carcinoembryonic antigen (CEA), and maximum and minimum standardized uptake value (SUV) of the lesion to discern ADC from SCC in a cohort of patients with stage III disease. From a training set of n=177 and validation set of n=78, they found an AUC of 0.87 (40).

#### 2.1.2 Morphologic subtypes within adenocarcinoma

Radiomic modeling can also be used to detect ADC morphologic subtypes (41–43), even among heterogenous tumors containing parcels of different morphologies. Chen et al. used radiographic data from CT images of 103 ADC with confirmed “near-pure” subtypes (defined as > 70% of a singular subtype) to evaluate ADC tumors with known heterogeneity to predict the presence of high-grade histology of micropapillary or solid patterns. In the validation set of heterogenous tumors, they found a sensitivity and specificity of 100% and 95.4% (44). To detect the presence of high-grade components (defined by micropapillary or solid morphology comprising ≥ 1% of the pathology report) in stage IA ADC, Pu et al. constructed a clinical-radiomic model from a cohort of n = 249 that incorporated both tumor and peri-tumoral features derived from CT images that was found to have an AUC of 0.85 and 0.82 on the internal and external validation sets (45). Huang et al. used a fusion model integrating radiomic features, clinical features, and intra-tumoral pixel clustering features to predict high-grade components in stage I ADC with an external validation AUC of 0.88 (46). Similarly, to recognize poorly differentiated histology for invasive ADC, Duan et al. integrated different peri-tumoral areas on CT scans into the region of interest in a combined clinical-radiomic model and found optimal AUC’s of 0.91 and 0.77 for the internal and external validation cohorts (47). Including both stage I and higher ADC, other research groups have been were able to construct a clinical-radiomic models to detect micropapillary and solid histological patterns (AUCs ranging from 0.78-0.94) (48–51). Using a combined clinical-radiomic model integrating ^18^F-FDG PET based features, Luo et al. and Lan et al. could identify high risk patterns (external AUCs = 0.83, 0.82, respectively) (52, 53). On the opposite end of the spectrum, radiomic modeling has shown encouraging ability to similarly identify lower grade and less aggressive tumors. Sun et al. analyzed patients undergoing resection with ground glass nodules (GGNs), and they found their radiomics and deep-learning based fusion model could differentiate MIA vs. IAC in GGNs with an AUC of 0.90 (54). Zhang and colleagues generated intra-tumoral heterogeneity scores for stage I ADC presenting as pure GGNs using pixel clustering and distribution, and their model was significantly better at identifying lepidic-predominant subtypes when compared to both conventional radiomic modelingand clinical radiological findings (AUC of the validation set 0.80 vs. 0.76 vs. 0.75 respectively) (55). Also looking for lepidic predominant subtypes in patients with pure GGNs, Zou et al. used a gradient boosting machine learning model in a cohort of 151 with both tumoral and peritumoral parenchymal radiomic features (AUC 0.96) (56).

#### 2.1.3 Spread through airspaces

Beyond just the histological subtype, radiomic markers have been shown to be adept at other pathological features of NSCLC that are associated with a more aggressive phenotype. Multiple radiomic models have been constructed to identify spread through airspaces (STAS) for ADC, a known aggressive feature (57). One study identifying STAS in stage I ADC by Chen et al. using just radiomic features in the model found an AUC 0.69 within a validation cohort of 122 (58). Building on this, two studies by Wang et al. and Chen et al. both constructed CT-based clinical-radiomic models for recognizing STAS in stage I ADC. AUC in the external test sets (n = 77 and n = 96) for both models were 0.89 and 0.88, respectively. Of note, both combined models had better fit than either models using just clinical or radiomic factors alone (59, 60). of Suh and colleagues similarly constructed a combined model to detect STAS in stage IA ADC; however, from their CT-based radiomic model, they calculated a numerical radiomic score “Rad-score” from the presence of significant features. This Rad-score, when integrated in combined predictive model with clinical factors, had a significantly better AUC than a clinical-only model on an external validation set of patients (n = 91) diagnosed with ADC one year after those included in the training set (AUC 0.83 vs. 0.74). The Rad-score was significantly higher in patients with STAS and with more aggressive morphologic subtypes, and a higher score was associated with worse five-year RFS on Kaplan-Meier survival curve (61). The above models all used CT imaging, but PET can also be used for detect STAS. Both Zheng et al. and Chen et al. generated radiomic-clinical models for identifying STAS in clinical stage I ADC patients using ^18^F-FDG PET/CT that had AUC’s in their respective validation cohorts (n = 40 and n = 64) of 0.898 and 0.92 (62, 63).

#### 2.1.4 Visceral pleural invasion and vascular invasion

Other pathological features that are characteristic of higher risk tumors have similarly been the subject for radiomic predictive modeling. Several studies have examined the use of clinical-radiomic models to identify visceral pleural invasion (VPI), which per the 9^th^ edition of International Association of the Study of Lung Cancer (IASLC) TNM classification, automatically qualifies the tumor as stage T2, regardless of tumor size (64). Five studies, all using CT imaging and a combination of clinical factors, clinical radiological observations, and radiomic extraction all found a combined model was a better predictor than radiomic or clinical modeling alone (AUC’s of validations sets were 0.81 – 0.89) (65–69). Of note, all used a pleural tag sign, pleural indentation sign, or pleural-tumor contact as a clinical radiographic predictor in the modeling; however, for all datasets, not all patients with pathologically-confirmed VPI were positive for such a sign, and some patients who did not have VPI did have that sign on imaging. This underlines the importance of not relying on a singular predictor for such pathological findings. Furthermore, Zhao et al. tested the performance of both a junior and senior radiologist who were blinded to pathological results in detecting VPI from CT images in clinical stage IA ADC with and without the aid of the model. They found the AUC, sensitivity and specificity of the junior radiologist improved from 0.61, 0.30, and 0.77 to 0.75, 0.36 and 0.90 respectively with the aid of the model. The senior radiologist also improved their AUC, sensitivity, and specificity from 0.73, 0.41, and 0.88 to 0.89, 0.64, and 0.91, all in the external test set (n = 160), highlighting the clinical applicability of the model (67). ^18^F-FDG PET/CT has also been used in modeling for VPI. Two studies with resectable NSCLC using combined models with clinical and PET radiographic modeling had AUC’s of 0.85 and 0.86 in the external validation sets (n = 99 and 160) (70, 71). Deng et al. examined microvascular invasion (MVI) in 188 stage I NSCLC patients using a combined model and found an AUC of 0.89 in the validation cohort (72). Though not an IASLC criterion for upstaging, MVI has shown to be an independent predictor for recurrence, and T1 carcinoma with MVI has been found to have comparable recurrence rates to T2 disease (73). Looking specifically at lympho-vascular invasion, Xu and colleagues analyzed 349 T1 ADC patients using habitat radiomic model which quantifies tumor heterogeneity by clustering sub-regions of voxels with similar characteristics, and their model had an AUC of 0.94, outperforming a deep learning model (AUC 0.90) (74). See **Table 1**, for a summary table of the above key studies using radiomic modeling to recognize histological patterns.

**Table 1:** Key studies on the use of radiomics for understanding tumor histology pre-treatment.

Authors	Year	Study type	Inclusion criteria	Study size	Key findings^a^
Panchawagh et al. (38)	2024	Retrospective imaging archive analysis	NSCLC patients with CT scans available in The Cancer Imaging Archive	N = 379 80:20% training:test ratio for machine learning 90:10% training: test for deep learning	Machine learning CT-based radiomic models can differentiate ADC, SCC, large cell, and undifferentiated histological NSCLC subtypes (AUC = 0.93)
Zhu et al. (39)	2018	Retrospective single-center cohort	Patients with resected ADC or SCC, excluding those with neoadjuvant therapy	Training (n = 81) Validation (n = 48)	Radiomic modeling of pre-operative CT imaging can differentiate ADC vs. SCC (AUC 0.89)
Zhang et al. (40)	2024	Retrospective single-center cohort	Patients with pathologically confirmed stage III ADC or. SCC	Training (n = 177) Validation (n = 78)	A combination model of clinical factors, and PET/CT-based radiomics can discern ADC from SCC. (AUC = 0.87)
Zheng et al. (41)	2025	Retrospective single-center cohort	Patients with resected non-mucinous ADC who did not receive neoadjuvant therapy	Training (n = 370) Validation (n = 159)	Radiomic based models could predict both >20% high-grade morphological patterns as well as the predominant subtype (AUC = 0.96)
Chen et al. (44)	2021	Retrospective single-center cohort	Patients with resected ADC and confirmed histological subtype	Training (n = 103) Validation (n = 55)	Combined clinical-radiomics model with CT imaging can predict micropapillary or solid morphological patterns in heterogenous tumors (sensitivity = 100.0%, specificity = 95.4%)
Pu et al. (45)	2025	Retrospective two-center cohort	Patients with resected stage IA ADC	Training (n = 228) Internal validation (n = 97) External validation (n = 80)	CT-based radiomic using tumoral and peri-tumoral features can detect high-grade features (AUC = 0.82)
Xie et al. (48)	2025	Retrospective single-center cohort	Resected stage I-IIA ADC	Training (n = 126) Validation (n = 54)	Combined CT radiomic-clinical model can detect high grade morphological features (AUC = 0.94)
Luo et al. (52)	2025	Retrospective single-center cohort	Confirmed invasive ADC	Training (n = 143) Validation (n = 60)	A combination CT-PET-clinical based model had was superior in detecting high risk morphological patterns (AUC = 0.86)
Sun et al. (54)	2025	Retrospective single-center cohort	Patients with resected GGN which were confirmed as MIA or IAC	Training (n = 177) Validation (n = 75)	Radiomic and deep learning fusion models can detect MIA vs. IAC (AUC = 0.90)
Zhang et al. (55)	2024	Retrospective two-center cohort	Patients with resected cT1 ADC presenting as pure GGN	Training (n = 429) Validation (n = 184)	Using intra-tumoral heterogeneity scores from CT based radiomic modeling, lepidic-predominant subtypes were recognized (AUC = 0.80)
Wang et al. (59)	2025	Retrospective multicenter cohort	Patients with resected stage I AC	Training (n = 226) Test set A (n = 306) Test set B (n = 77)	CT based clinical-radiomic model can identify STAS (AUC = 0.89)
Suh et al. (61)	2024	Retrospective single-center cohort	Resected stage IA ADC	Training (n =211) Internal validation (n = 91) Temporal validation (n = 158)	CT-based radiomic model to create a “Rad-score” which was associated with STAS (AUC = 0.83) and associated with worse RFS
Zhao et al. (67)	2025	Retrospective two-center cohort	Resected cStage IA ADC	Training (n = 289) Validation (n = 160)	CT-based deep learning-clinical-radiomic model could identify VPI (AUC 0.81). Aid of the model helped radiologist recognition of VPI
Li et al. (71)	2025	Retrospective multicenter cohort	Resected NSCLC with preop PET/CT with metabolic activity	Training (n = 228) Internal validation (n = 97) External validation (n = 80)	Combined PET/CT radiomic-clinical model can detect VPI. (AUC = 0.86)
Deng et al. (72)	2024	Retrospective single-center cohort	Stage I NSCLC	Training set (n = 133) Validation set (n = 55)	Combined clinical-radiomic based model could detect microvascular invasion (AUC = 0.79)
Xu et al. (74)	2025	Retrospective multi-center cohort	T1- stage ADC	Training set (n = 210) External validation set (n = 139)	Habitat-derived radiomic model could detect lymphovascular invasion (AUC = 0.94)

NSCLC, non-small cell lung cancer; ADC, lung adenocarcinoma; SCC, lung squamous cell carcinoma; AUC, area under the curve; AIS, adenocarcinoma-*in-situ*; MIA, minimally-invasive adenocarcinoma; IAC, invasive adenocarcinoma; GGN, ground glass nodule; STAS, spread through air spaces; VPI, visceral pleural invasion.

^a^ AUC values provided are all from validation sets where able.

### 2.2 CANARY radiomics and tumor aggressiveness

**Figure 2:**
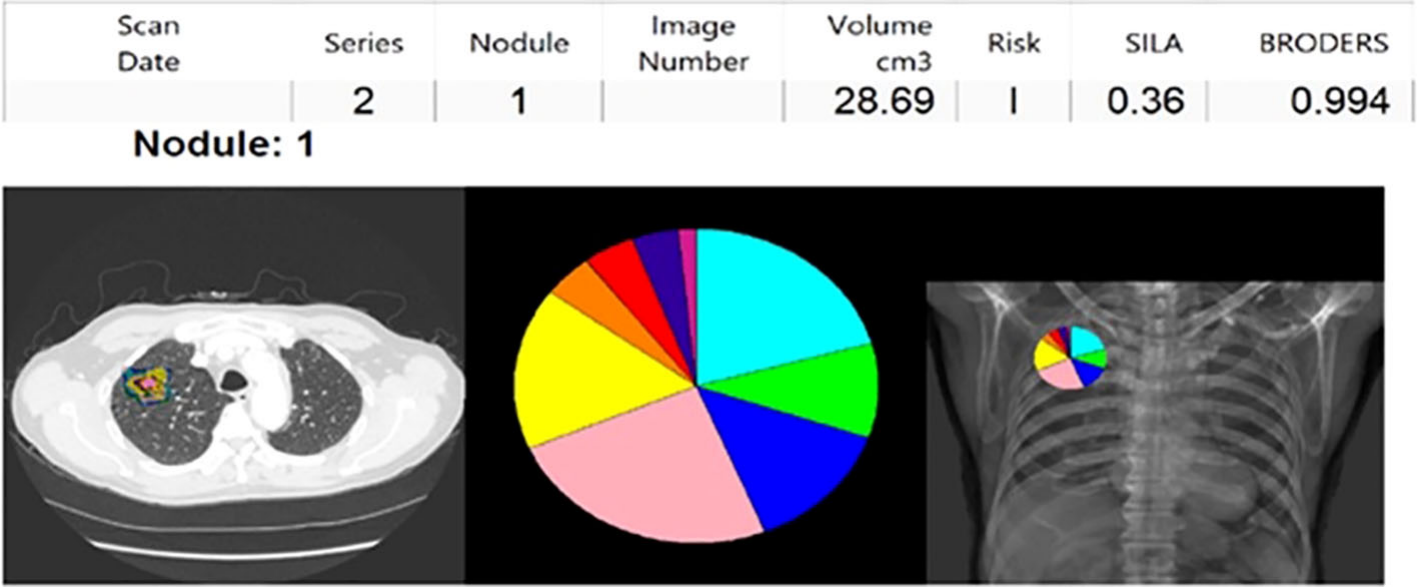
Example annotation, exemplar distribution, and Score Indicative of Lung Cancer Aggression (SILA) after Computer-Aided Nodule Assessment and Risk Yield (CANARY) analysis. Image adapted from Park et al. under the Creative Commons Attribution-NonCommercial-NoDerivs 4.0 International License (CC BY-NC-ND 4.0 https://creativecommons.org/licenses/by-nc-nd/4.0/.) DOI: 10.21037/jtd-24-923 (78).

One radiomic software tool that has been developed to generate prognostic risk score is the Computer-Aided Nodule Assessment and Risk Yield (CANARY) software. Several studies have been published charting its use to risk stratify NSCLC. CANARY was initially developed as a radiomic tool to classify tumor aggressiveness by segmenting out the nodules within the training set images and then further subdividing the segments into regions of interest (ROI) within the defined nodule area. These ROI were then clustered and classified as one of nine color-coded exemplars. From the training set, the exemplars were secondarily clustered into one of three groups (low-, intermediate-, or high-risk) to affiliate with a relative degree of tumor aggressiveness, with aggressive tumors defined as IAC and indolent as AIS or MIA. In the test set, each voxel was compared against the nine exemplars and assigned to the most similar one (75). For the feasibility pilot study, the use of CANARY to identify aggressive vs. indolent ADC within a cohort of n = 86 had a sensitivity of 98.7%, specificity of 63.6%, positive predictive value of 94.9% and negative predictive values of 87.5%. When assessing the degree of histopathological invasion (on a scale of 0-100%), the CANARY estimated % invasion had very close correlation with the pathological findings (validation set Spearman R = 0.89, p < 0.0001) (75). Part of CANARY’s clinical applicability is because it operates as a semi-automated software that requires the user to select the nodule of interest. It then generates a “mask” outlining the borders of the nodule for each CT image slice, and the user can subsequently manually refine the borders. Once confirmed, CANARY automatically assigns exemplars to each voxel, generates a risk score, and classifies the nodule (**Figure 2**) (76).

CANARY has since been validated and utilized by multiple research groups to evaluate tumor aggressiveness and prognosis. Nemec et al. validated the software by independently applying CANARY to 64 pure GGNs with pathologically confirmed ADC, and the relative volume percentage belonging to each of the three low, intermediate, or high-risk exemplar groups was significantly different between the histological subtypes of AIS, MIA, and IAC, i.e. the volume of low-risk components was significantly more in AIS than MIA or IAC nodules and intermediate- and high-risk volumes were significantly more in IAC than MIA and AIS. Furthermore, the degree of invasive focus on pathological examination was significantly associated with the volume of intermediate and high-risk components (77). The initial research group used CANARY to generate the Score Indicative of Lung Cancer Aggression (SILA) which is an aggregate of the normalized distributions of the ordered exemplars and their relative risk profiles, and it is presented as a value on a continuous scale from 0 to 1. In a cohort of 237 resected lung ADC specimens, they found the SILA was able to differentiate indolent vs. invasive tumors (AUC 0.91, p < 0.001), and the SILA increased with the pathologically confirmed depth of invasion (79). Lee et al. then validated the use of SILA in a Korean population of 380 GGNs and found an AUC of 0.81 in predicting indolent vs. invasive tumors, though a lower optimal predictive threshold than the Mayo group for SILA had to be used (0.229 vs. 0.338) (80). Steiner and colleagues, using a cohort of 161 resected known stage I ADC specimens, correlated an increasing SILA positively with invasive size at resection (*R* = 0.54, p = 8.0×10^-14^) and negatively with the percentage of lepidic histology (*R* = -0.46, p = 7.1×10^-10)^. Furthermore, the SILA was also significantly associated with the presence of vascular invasion, even after controlling for invasive size (81).

These studies across different patient populations highlight the applicability of the software, and the semi-automated nature of its use lends CANARY genuine potential for integration into clinical practice. After analysis of the nodule, CANARY automatically generates a SILA score. A table highlighting the studies investigating CANARY and the evolution of its usage is provided in **Table 2**. The same research group which developed CANARY has developed a quite similar tool to differentiate benign vs. malignant nodules from incidentally discovered indeterminant nodules (82). Between the two, these models offer the ability for a near virtual biopsy to determine malignancy and then offer insights on the underlying aggressiveness of a possible carcinoma.

**Table 2:** Key studies using CANARY for radiomic analyses.

Authors (ref.)	Year	Inclusion criteria	Study size	Key findings
Maldonado et al. (75)	2013	Resected ADC nodules	Training set n = 86 nodules; Test set n = 54 nodules	Development of CT-based CANARY. CANARY could predict invasive vs. indolent ADC (sensitivity = 98.7%, PPV = 94.4%) and percentage of invasive component.
Raghunath et al. (84)	2014	Resected ADC nodules ≤ 3cm in size	N = 264	CANARY stratifies patients into three “good”, “intermediate”, and “poor” risk groups based on coded exemplars. The risk groups are significantly associated with 5-year RFS
Clay et al. (85)	2017	Resected ADC nodules	N = 118	Yellow and green exemplars are associated with EGFR mutations.
Nemec et al. (77)	2018	Resected ADC nodules presenting as pure GGN	N = 64	Percentage volume from each exemplar group in nodules was significantly different between AIS, MIA, and IAC
Nakajima et al. (86)	2018	CT images of confirmed ADC	N = 95	Low inter-operator variability between CANARY users when segmenting nodule volumes
Varghese et al. (79)	2019	Resected ADC nodules	N = 237	Development of SILA as a numerical risk score based on the aggregate of the exemplars. SILA is able to differentiate invasive vs. indolent tumors and increases with depth of invasion. SILA risk scores are also correlated with DFS.
Lee et al. (80)	2021	Resected ADC-confirmed GGNs	N = 380	SILA can be used to predict invasive vs. indolent tumors, but a different predictive threshold was used from Varghese et al.
Al-Ghoula et al. (76)	2023	CT images of confirmed ADC	N = 95	Low inter-operator variability in nodule segmentation and SILA scores across user training levels
Park et al. (78)	2024	Patients with resected ADC ≤ 2cm	N = 134	“Poor” classification by SILA risk score was associated with increased risk of recurrence
Pham et al. (87)	2024	Patients with resected ADC clinical stages IA-IIA	N = 228	CANARY SILA scores were significantly associated with OLNM. “Poor” classification was associated with higher rates of OLNM
Steiner et al. (81)	2025	Resected ADC stage I nodules	N = 161	Increasing SILA scores are associated with increasing invasive size and likelihood of vascular invasion as well as negatively correlated with the percentage of lepidic histology

CANARY, computer-aided nodule analysis and risk yield; ADC, lung adenocarcinoma; PPV, positive predictive value; RFS, recurrence-free survival; GGN, ground glass nodule; AIS, adenocarcinoma-*in-situ*; MIA, minimally invasive adenocarcinoma; IAC, invasive adenocarcinoma; SILA, Score Indicative of Lung Cancer Aggression; DFS, disease-free survival; OLNM, occult lymph node metastasis.

### 2.3 Radiomics as a prognostic tool

Based on current practice guidelines, the gold standard management for patients with an acceptable risk level with early-stage lung cancer remains a surgical resection (83). Yet for these patients, it is still a difficult challenge to reliably prognosticate their post-operative outcomes in terms of tumor progression. As a result, radiomics has been increasingly investigated in order to provide additional data points about tumor characteristics and individualized surgical and medical management plan at the pre-operative stage.

Some radiomic-based predictive models have been packaged and are ready-made for clinical use by others. One such tool that has been validated based on surgical samples is CANARY. As part of the development of CANARY, Raghunath et al. collected a cohort of 264 surgically resected lung ADC nodules ≤ 3cm and applied CANARY to their pre-operative high-resolution CT imaging. Using the aggregate CANARY scores based on the nine distinct exemplars, they were able to devise three distinct and natural clusters of the exemplars. Each nodule was then classified into one of the clusters. The three groups then had their respective 5-year disease free survival (DFS) compared which resulted in 100%, 72,7% and 51.4% DFS for each group (log-rank test p = 0.0005) (84). These groups of good, intermediate, and poor risk would go on to become the basis for SILA as the relative percentage of each exemplar from each group would confer a relative degree of risk (79). Varghese et al. subsequently assigned SILA scores to good, intermediate, and poor survival groups based on pathologically-confirmed depth of invasion (AIS/MIA vs. depth of 6-20mm vs. depth > 20mm). They found significant difference in Kaplan-Meier survival curves based on depth (100% vs. 76% vs. 60% 5-year DFS) but also found similar survival with plotting survival curves differences based on the SILA scores (100% vs. 79% vs. 58% 5-year DFS) (79). In a study to investigate the association between CANARY exemplars and genetic mutations, Clay et al. found that no patients with TP53 mutations were associated with the “good” prognosis exemplar group, and all tumors with EGFR and TP53 co-mutations were classified as intermediate by CANARY (85).

Based on these promising data from CANARY, other research groups have applied its analysis to their own patient samples. Park et al. examined its role in risk stratification for clinical stage I ADC with nodules ≤2 cm in size. In a sample of 134 patients undergoing resection, 29 (21.6%), 52 (38.8%), and 53 (39.6%) were categorized as good, intermediate, and poor respectively by their SILA scores. There was also a statistically significant difference in terms of outcomes, with their 3-year RFS for the good, intermediate, and poor patients at 96.3%, 92.0%, and 72.7%, respectively (p = 0.002) (78). With new evidence showing that outcomes for patients with peripheral stage I ADC with a sub-lobar resection is noninferior to those receiving a lobectomy, these observations from Park et al. pointed towards the fact CANARY can play an important role in identifying more aggressive nodules even prior to surgery. Patients with poor CANARY risk then can be further engaged in the decision of the extent of their resection.

Beyond pre-packaged radiomic tools that operate as all-in-one programs to extract features from the nodule, analyze the feature data, and then provide the risk score, other tools have been developed to facilitate and help standardize one step in the radiomics workflow. One such tool is PyRadiomics. PyRadiomics is a freely available open-source software (https://pyradiomics.readthedocs.io) that is based on the Python coding language and can be used to extract radiomic features from a variety of medical imaging modalities (e.g. CT, MRI, PET) (88). PyRadiomics is one of the most popular software packages to extract the feature data that can be used for statistical analyses for the study question of interest. The features extracted follow the Image Biomarker Standardization Initiative (IBSI) features list which is a list of standardized radiomic features that are extracted, aiding reproducibility across studies (89). PyRadiomics is not specific to the use of radiomics to investigate NSCLC. It is a generalized feature extraction tool that is used across many radiomics studies investigating diverse outcomes of interest. It has served as a base extraction software for several radiomics studies for NSCLC and other unrelated benign and malignant disease processes. It does not perform the risk modeling like CANARY or perform a statistical comparison of between the feature data. It instead just processes the images and provides the quantitative data that can later be used in feature selection and then modeling.

One of the major benefits of using such a popular and open-source software to aid in a radiomics-based study is that it provides a standardized feature library for extraction and a standardized process for extraction. Therefore, the same features may be used across different studies, and they will be extracted by the same software. Although the exact features extracted and some settings for extraction may be customized (an overview of customizable settings available at https://pyradiomics.readthedocs.io/en/latest/customization.html), a base program for feature extraction across multiple studies offers some standardization in methodology for deriving the radiomic data, even if the statistical modeling may be different. PyRadiomics is only one of several different radiomic feature extraction programs available, and different studies may select different programs that best fit with their workflow and institutional operating systems. Due to its popularity, it is singled-out as a platform for discussion in this review to illustrate how using a list of standardized features extracted by the same tool can be used to investigate a variety of different outcomes which aids in reproducibility across methodologies. On its own, it does not provide any usable clinical information for decision-making. But by using a widely-available software with reproducible feature extraction, researchers know there is commonality between the studies and the underlying data from which the resultant models are derived.

PyRadiomics has been used in numerous NSCLC studies which have shown that the extracted radiomic feature data can then be used for disease course prognostication after statistical modeling. In a sample of 293 patients of surgically resected clinical stage IA-IIIA NSCLC, Ho et al. applied PyRadiomics and developed four different models to assess recurrence risk: radiomic-clinical, radiomic, clinical, and TNM stages. They found that the combined radiomic-clinical model performed better the other three with an AUC of 0.77. In comparison, the radiomic, clinical and TNM stage models had an AUC of 0.76, 0.71, and 0.70, respectively. Moreover, stratification based on the radiomic features into high-risk and low-risk group yielded a significantly higher recurrence risk within the high-risk group (p < 0.01) (90). In a separate analysis, Choe et al. further strengthened the case for radiomics signature gathered from PyRadiomics being effective in prognostication. Among 1058 patients with clinical stage IA-IIIA lung ADC, DFS and OS were significantly associated with two distinct sets of radiomics features. Additionally, while the radiomics signatures model performed similarly to the clinical-pathologic model in the dataset, the combined clinical-pathologic-radiomics model was the best performing with a C-index of 0.782 (p = 0.002) and 0.805 (p = 0.006) for DFS and OS, respectively (91). In a similar set of 234 patients with clinical stage I-IIIA lung ADC across multiple centers, Liu et al. used PyRadiomics to pull radiomic features which were then ranked in accordance with its association with OS. These features were then ranked, paired and tested against OS to determine the significantly associated pairs. 12 radiomic feature pairs were identified as significant, and from these a risk score was calculated. A time-dependent ROC curve was used to determine an optimal risk score cut-off to classify patients as high or low risk. In the external validation set, they found that high-risk patients with the radiomic signature had significantly shorter OS (hazard ratio [HR] 10.5, 95% confidence interval [CI] 1.36 – 81.59, p = 0.005, C-index = 0.73). The radiomic signature had an AUC of 0.74 for modeling 5-year OS, and multivariate Cox analysis showed independent prediction of OS, even with controlling for clinical factors such as TNM stage, grade, lymphovascular or pleural invasion, surgery type, and histological subtype (92). Wang et al. built a clinical-radiomic model for a cohort of 152 stage IA – III resected NSCLC patients (training set n = 121, test set n = 31) using 29 distinct radiomic features and the N stage to associated with postoperative recurrence (AUC 0.94 in test cohort) (93). Building a combined model with IASLC grading criteria and radiomic features in Stage IA ADC, Chen et al. found a radiomics signature significantly associated with OS and disease-free survival and a combined model AUC of 0.79 at 5-year OS (94).

To refine the extent of peri-tumoral parenchyma to assess, Tominaga and colleagues analyzed PyRadiomics extracted features from gross tumor and peri-tumoral volumes at 3, 6, and 9 mm boundaries from the tumor to construct and compare models. From a cohort of 526 patients (external validation set n = 121) with surgically resected clinical stage 0-IIB NSCLC, they found the model with the gross tumor and 3 mm peri-tumoral parenchymal boundaries performed the best and significantly outperformed just the gross tumor model in the training, internal, and external validation cohorts in association with OS (external cohort C-index = 0.71) (95). Wang et al. even further restricted the use of PyRadiomics to a cohort of pure solid pathologic stage IA NSCLC and found similar success. From 592 patients, with 381 in the training cohort, 163 in the internal validation cohort, and 48 in the external validation cohort, they extracted three distinct regions of interest: intratumoral 2-D region, intratumor 3-D volume, and peritumoral area. They then combined these three regions into a singular radiomic signature that achieved a 3-year and 5-year AUC for modeling RFS of 0.76 and 0.75, respectively, in the external validation set. Moreover, in the multivariable analysis, this radiomic signature was found to be an independent prognostic factor for OS (p<0.001) (96).

Deep learning algorithms have also been used to extract radiomic features for prognostication. Deep learning uses multiple layers of neural networking to process large amounts of data to extract feature data, recognize feature patterns, and create a feature signature without manual delineation of features. The features identified in deep learning are not selected by the users but are instead identified through the multiple layers of pattern recognition. Kuang et al. utilized a cohort of 459 patients with pathologically confirmed solid-nodular stage I NSCLC and applied a deep learning feature extraction program (ResNet-18) to identify a radiomic feature signature associated with prognosis. They ultimately found that when they combined the identified deep learning radiomics signature with clinical and pathological factors such as size, pathological stage, and carbohydrate antigen 19-9, this integrated model was a better indicator for of post-operative disease progression than any of the other factors alone (97). Using resected stage I-III NSCLC, Hosny et al. used a deep learning network- based model to predict 2 year OS with an AUC of 0.71 (98). A summary table of the key studies investigating radiomic-based models and prognosis is provided in **Table 3**.

**Table 3:** Key studies on the use of radiomics in prognostication.

Authors	Year	Study type	Inclusion criteria	Study size	Key findings^a^
Raghunath et al. (84)	2014	Retrospective single-center cohort	Resected ADC nodules ≤ 3cm in size	N = 264	CANARY stratifies patients into three “good”, “intermediate”, and “poor” risk groups based on coded exemplars. The risk groups are significantly associated with 5-year RFS
Varghese et al. (79)	2019	Retrospective single-center cohort	Resected ADC nodules	N = 237	Development of SILA as a numerical risk score based on the aggregate of the exemplars. SILA is able to differentiate invasive vs. indolent tumors and increases with depth of invasion. SILA risk scores are also correlated with DFS.
Park et al. (78)	2024	Retrospective single-center cohort	Patients with resected ADC ≤ 2cm	N = 134	“Poor” classification by SILA risk score was associated with increased risk of recurrence
Ho et al. (90)	2025	Retrospective single-center cohort	Resected cStage IA-IIIA NSCLC	Training (n = 195) Validation (n = 98)	A combined PyRadiomics CT radiomic-clinical model was superior in predicting 3 year recurrence and could classify patients into high and low recurrence risk groups
Choe et al. (91)	2025	Retrospective single-center cohort	Resected cStage IA-IIIA	Training (n = 754) Validation (n = 304)	PyRadiomics generated CT radiomic-clinical-pathologic model was predictive of DFS and OS
Tominaga et al. (95)	2024	Retrospective single-center cohort	Resected cStage 0-IIB NSCLC	Training (n = 191) Internal validation (n = 160) External validation (n = 175)	Integration of PyRadiomics features from peri-tumoral parenchyma improved radiomic model prediction of OS (C-index = 0.71)
Wang et al. (96)	2024	Retrospective single-center cohort	Resected pure-solid clinical and pathologic stage IA NSCLC	Training (n = 381) Internal validation (n = 163) External validation (n = 48)	Radiomic signature from PyRadiomics generated features was predictive of RFS (AUC = 0.75) and associated with OS
Kuang et al. (97)	2024	Retrospective single-center cohort	Resected pStage I NSCLC	Training (n = 321) Internal validation (n = 138) External validation (n = 104)	Deep learning generated radiomic signature when combined with clinical factors was predictive of post-op disease progression (AUC = 0.86)

ADC, lung adenocarcinoma; CANARY, computer-aided nodule analysis and risk yield; RFS, recurrence-free survival; SILA, Score Indicative of Lung Cancer Aggression; NSCLC, non-small cell lung cancer; DFS, disease-free survival; OS, overall survival; RFS, recurrence-free survival; AUC, area under the curve.

^a^ AUC values provided are all from validation sets where able.

### 2.4 Radiomics and detection of occult lymph node metastasis

The topic of occult lymph node metastases (OLNM) is a major point of interest in the potential application of radiomics. By definition, these metastases can only be reliably discovered after resection, which will then upstage the patient and change both the management plan and prognosis. Radiomic based studies have endeavored to determine which subsets of patients might be at a higher risk for OLNM or develop models that can estimate risk in order to better inform surgeons at the preoperative stage (99, 100).

CANARY has been enlisted to determine an association between its generated SILA risk scores and OLNM. Pham et al. collected 228 patients with clinical stages IA-IIA among whom 28 (12.3%) patients had pathologically proven occult lymph node metastases. The breakdown of CANARY profiles for these patients were 1 (3.6%), 3 (10.7%), and 24 (85.7%) with good, intermediate, and poor risks, respectively. There was a statistically significant association between their SILA scores and associated poorer prognosis and occult lymph node metastases (87). This work further strengthened the growing body of data on CANARY being able to non-invasively stratify the risk of recurrence, particularly for patients with nodules deemed to be poor risk.

PyRadiomics has also been used as the extraction tool in studies seeking to associate radiomic features with OLNM. Applying the tool to CT scans of a training dataset of 880 patients and a validation dataset 322 patients with surgically resected clinical stage I ADC and who underwent systemic mediastinal lymph node dissection, Yang et al. identified five radiomic signatures of extracted features that had a statistically significant association with N2 disease: energy, skewness, maximum probability, joint entropy, and gray level variance and two clinical factors: tumor size and CEA. In the validation set, AUC’s for the clinical model, radiomic model, and combined model were 0.64, 0.82, and 0.83, respectively. The identified radiomics signature showed better performance than clinical factors (p < 0.001), but there was no significant difference between the radiomic vs. combined models (p = 0.23) (101).

Other research groups have integrated deep learning algorithms to detect OLNM. Zhang et al. enrolled 140 clinical stage I-II NSCLC patients to apply radiomics features analysis with deep learning models to improve N2 detection. They compared a ResNet-18 deep learning architecture with a traditionally constructed radiomics signatures using three sets of features: C1 (original features), C2 (Laplacian of Gaussian filtered), and C3 (wavelet-transformed). Even though the performance of the traditional radiomics-only model was not statistically different when compared to the deep learning-only model, they found the best N2 metastases identification came from a combined model of radiomics, deep learning, and clinical factors with an AUC of 0.88 (102). Also using ResNet18 neural networking, Tian et al. examined a cohort of 1325 clinical T1aN0 solid predominant invasive NSCLC patients to predict OLNM. They found the fusion radiomics-deep learning model pre-trained with concatenation was the best identifier with an average AUC across 3 validation and test sets of 0.75 (103). Huang et al. also used deep learning based radiomic modeling. From 165 patients with pathologically confirmed lymph node metastasis out of 1099 patients undergoing resection for adenocarcinoma, the authors found that a combined model using both deep-learning derived radiomic signatures with clinical factors had superior performance in detecting OLNM over either a clinical or just deep-learning based model individually (104). Xia et al. tackled the same problem with a cohort of 724 patients with early-stage invasive lung ADC. They were able to identify 6 key radiomics features that were associated with occult lymph node metastases along with several clinical factors. After integration with a deep-learning algorithm, the combined model was statistically superior in recognizing lymph node metastasis over a clinical or pure radiomic model (independent test set AUC’s 0.91 vs. 0.84 vs. 0.85 respectively, p < 0.05) (105). The use of a combined model for identifying lymph node metastasis in clinical stage I NSCLC was echoed by the findings of Duan et. al., which used deep learning integration on the analysis of PET/CT imaging and Xie et. al., which integrated deep learning with standard radiomic extraction CT imaging (106, 107).

One other effort included the use of an artificial intelligence (AI) automated program in the analysis of CT scans for clinical stage 0-IA NSCLC to identify nodal metastases. Shimada et al. maintained a database of 720 patients that underwent complete resection. They used the AI software Beta Version which automatically detects and segments the lung nodules, reconstructs a 3D image, and uses a convolutional neural network to extract radiomic features. They found the AI analysis computed an average solid-CT value score for each tumor which was independently associated with pathologically confirmed lymph node invasion and unfavorable RFS on multivariate analysis (p=0.033) (108). A table of the studies investigating the ability of radiomic models to identify lymph node metastasis is provided in **Table 4**.

**Table 4:** Use of radiomics to detect occult lymph node metastases pre-operatively.

Authors	Year	Study type	Inclusion criteria	Study size	Key findings^a^
Pham et al. (87)	2024	Retrospective single-center cohort	Patients with resected ADC clinical stages IA-IIA	N = 228	CANARY SILA scores were significantly associated with OLNM. “Poor” classification was associated with higher rates of OLNM
Yang et al. (101)	2019	Retrospective single-center cohort	Patients with resected stage I ADC	Training (n = 880) Validation (n = 332)	Model using PyRadiomics extracted radiomic features outperformed a clinical model to detect N2 metastasis (AUC = 0.83)
Zhang et al. (102)	2023	Retrospective single-center cohort	Patients with clinical stage I-II NSCLC	Training (n = 98) Validation (n = 42)	A combination model of Multiview radiomics, deep learning, and clinical features can predict pre-surgical N2 disease. (AUC = 0.88)
Tian et al. (103)	2024	Retrospective multiple-center cohort	Patients with solid-predominantly invasive ADC cT1a-bN0M0	Training (n = 470) Internal validation (n= 202) External validations (n_1_ = 227, n_2_ = 426)	Combined radiomics and deep learning model can predict occult lymph node metastases from pre-operative CT scans (Average AUC = 0.75)
Huang et al. (104)	2025	Retrospective two-center cohort	Patients with resectable ADC	Training (n = 739) Internal validation (n = 318) External validation (n = 42)	2.5D deep learning radiomics when combined with clinical factors can predict OLNM (AUC = 0.90)
Xia et al. (105)	2025	Retrospective two-center cohort	Confirmed ADC ≤4cm without distant metastases and no neoadjuvant treatment	Training (n = 418) Internal validation (n = 106) External validation (n = 200)	Combined deep learning-radiomic-clinical model was superior in predicting lymph node metastasis (AUC = 0.91)
Duan et al. (106)	2025	Retrospective two-center cohort	Patients with resected ADC	Training (n = 136) Internal validation (n = 59) External validation (n = 53)	Clinical, radiomics, and deep-learning features derived from ^18^F-FDG PET/CT scans can be used to predict occult lymph node metastases (AUC = 0.87)
Xie et al. (107)	2024	Retrospective two-center cohort	Patients with resected ADC and pathologically confirmed lymph node status	Training (n = 401) Validation (n = 102)	Combined radiomics and deep learning models can predict lymph node status based on pre-operative contrast-enhanced CT scans (AUC = 0.85)
Shimada et al. (108)	2023	Retrospective single-center cohort	Patients with resected stage 0-IA NSCLC	Training (n = 480) Validation (n = 240)	Artificial intelligence-processed average solid CT attenuation value can predict pathological nodal metastases in early-stage NSCLC (AUC = 0.76)

ADC, lung adenocarcinoma; CANARY, computer-aided nodule analysis and risk yield; SILA, Score Indicative of Lung Cancer Aggression; OLNM, occult lymph node metastasis; AUC, Area under the curve; NSCLC, non-small cell lung cancer.

^a^ AUC values provided are all from validation sets where able.

### 2.5 Radiogenomics

Beyond just the phenotype of the underlying tumor, radiomics can potentially offer insight into the genomic alteration and resultant mutant protein expression of the disease before any biopsy is actually taken. Multiple oncogenic drivers have been linked to NSCLC such as epidermal growth factor receptor (*EGFR*), Kirsten rat sarcoma oncogene homologue (*KRAS*), and anaplastic lymphoma kinase (*ALK*), and mutations in these oncogenes have been linked to downstream effects in both treatment and prognosis (109–118). In resource-limited settings where full genomic panel testing may not be readily available or would be cost prohibitive, radiomic predictive modeling for oncogenic drivers and treatment targets would be quite valuable. Additionally, if biopsy tissue yields are small and inadequate for testing, having reliable, non-invasive predictive modeling would save patients from undergoing further invasive non-therapeutic procedures.

*EGFR* is one of the most common oncogenic drivers, and mutations in this gene offer unique treatment targets via tyrosine-kinase inhibitors (TKI) (112–114). Several studies have used CT imaging based clinical-radiomic models to link imaging findings with EGFR mutations (119–123). As TP53 co-mutation is known to negatively modulate responsiveness to EGFR-TKI’s (124), Wang et al., from a sample of 267 ADC patients, were able to first identify if a patient had an EGFR mutation (test cohort AUC 0.78) and then within that group if there was a TP53 mutation (AUC 0.80) (121). Zhang et al. compared using non-enhanced images, arterial phase, venous phase, or a combination of the three in their model. They found that for identifying an EGFR mutation, the multiphase model outperformed any of the three individual sequence model or a clinical factor model (AUC 0.93 vs. 0.86, 0.79, 0.75, 0.71 respectively) (123). Among patients with known EGFR mutation, Lu and colleagues were able to predict the presence of specifically a T790M mutation which is particularly common in NSCLC with acquired resistance to TKI (125–127). From a cohort of 274 patients with known EGFR mutation (validation cohort n = 82), their combined model had an AUC of 0.86 to predict T790M mutation (127). Multiple studies have also associated ^18^F-FDG PET/CT imaging with EGFR mutations (128–130). Using PET information from 150 ADC patients, Li et al., were able to construct a comparable clinical-radiomic model to detect EGFR-TP53 co-mutation (validation set AUC 0.79) (128). CANARY has also been used to identify EGFR mutation. Clay and colleagues found that in a combined model, smoking, low pulmonary fibrosis of the surrounding parenchyma and the presence of the yellow- or green-colored CANARY exemplars were associated with EGFR (AUC 0.87) in a cohort of 118 ADC cases (85).

*EGFR* is not the only oncogene that has been investigated using radiomics. Mahmoud and colleagues compared non-contrasted CT images and contrasted CT images from 815 ADC patients to construct a clinical-radiomic model associated with KRAS mutations. Within the validation set, the non-contrast CT model showed an AUC of 0.87, and the contrasted CT had an AUC of 0.88 (131). Chen et al. used both CT-based tumor radiomics features and peri-tumoral regions along with clinical factors to identify ALK mutations in a total cohort of 505 with 3 external validation sets (AUC in the 3 validation sets 0.81, 0.86, 0.88) (132). Similarly, another combined model to identify ALK mutations in cohort of 210 had an AUC of 0.80 (133). A meta-analysis by Fuster-Matazano et al. of 51 studies using CT based radiomic AI models to predict mutations in the EGFR, ALK, and KRAS oncogenes found overall AUC’s of 0.77, 0.83 and 0.73, respectively. Pooled sensitivities were 0.75, 0.75, and 0.48, respectively, though there was significant heterogeneity in the KRAS study results (134).

The above studies show that radiomic-based models can have strong potential for detecting the presence of mutated oncogenes. In resource-limited settings where genetic testing may not be readily available, these models can offer insight into the underlying genomic makeup of the tumor, and as such, they can help with making predictions about the tumor behavior and responsiveness to therapy.

### 2.6 Radiomics and PD-1/PD-L1 expression

Along with detection of oncogenic mutations, radiomic based models have also shown potential for identification of tumor proteomic expression. Multiple studies have particularly focused on the PD-1/PD-L1 axis expression, which is the basis for ICI therapy. With the integration of immunotherapy and increasingly widespread use of ICI for both resectable and non-resectable tumors, evidence of expression or lack thereof of programmed cell death-1 (PD-1) or programmed death-ligand 1 (PD-L1) has become vital. Especially as neoadjuvant chemo-immunotherapy has been shown to improve oncologic outcomes in resectable NSCLC (17), an understanding of protein expression pre-operatively is paramount. Using a total cohort of 200 patients, Tian et al. constructed a nomogram for PD-1 expression, and the combined clinical radiomic model was found to have an AUC of 0.88 and 0.80 in the internal and external validation cohorts (135). Using a deep learning model, Lu et al. constructed a similar combined model on 352 NSCLC patients associated with PD-L1 expression which exhibited an AUC of 0.91 in the validation set (136). Using specifically arterial phase enhanced CT images, Liu et al. used various radiomic modeling algorithms, and their random forest model was able to estimate PD-L1 non-expression (< 1%), low-expression (1-49%) and high expression (≥ 50%) with AUC in the validation cohort (n = 58) of 0.95, 0.93 and 0.94 respectively (137). Peng et al. constructed a combined machine learning model using ^18^F-FDG PET/CT to recognize PD-L1 expression which using an SVM algorithm had an AUC of 0.88 from a cohort of 143 patients (138). Using habitat analysis of metabolic activity on ^18^F- FDG PET/CT from 143 NSCLC patients, Ji et al. developed a radiomic model with an AUC of 0.79 in the test cohort. Furthermore, tumors with a relatively high percentage of habitat volume of high-glycolytic/high-density activity were positively correlated with PD-L1 expression (139).

These radiomic models and their ability to detect protein expression can be important when there are pathological detection limitations. Preoperative biopsies are not always particularly robust in the tissue yield. When the decision to administer neoadjuvant immunotherapy is based on the percentage of cells expressing a particular protein, a certain volume of tissue is required to be able to stain. Having adjunct models to aid in prediction for proteomic expression would be important in such cases where initial biopsies may be inadequate for staining. If a radiomic model is strongly suggestive, further invasive procedures and the accompanying risks might be avoided.

### 2.7 Radiomics and predicting response to therapy

Radiomics can predict responsiveness to treatment beyond just providing predictions for survival or odds of recurrence. The integration of neoadjuvant immunotherapy has shown oncological benefits in appropriately selected patients (17, 18, 20, 140). However, upon surgical resection after neoadjuvant therapy, a wide range of pathological responses have been noted with some patients experiencing complete response with no viable tumor cells and some with no response and tumor progression (17, 141–143). Therefore, there is a potential role for radiomics to help predict which patients might be stronger responders to ICI. Liu and colleagues used ^18^F-FDG PET/CT images from 210 NSCLC patients with PyRadiomics to build a clinical-radiomic model to predict pathologic complete response (pCR) to neoadjuvant ICI. They found the combined model which integrated radiomic features with histological type and clinical stage was significantly better at predicting pCR over the sole clinical or radiomic models (test set AUC 0.82 vs. 0.63 vs. 0.67, respectively, p < 0.05 for all) (144). Fan et al. used tumoral and peritumoral features from the pretreatment CT to predict pCR (AUC = 0.75), and Yang et al. used a combined PET and CT based model to predict pCR (AUC = 0.82) (145, 146). Not all studies have been as emphatic though. Using a sample of 211 patients with NSCLC, Wang et al. constructed a clinical-radiomic model to predict major pathologic response (MPR), defined as < 10% vital tumor tissue on surgical resection, using PyRadiomics extracted features from CT imaging and using clinical change in tumor size, which was determined based on the Response Evaluation Criteria in Solid Tumors (RECIST). In the validation cohort, the combined model outperformed the radiomic model (AUC = 0.80 vs. 0.60, p = 0.001), but the clinical model was comparable (AUC = 0.80) (147).

Delta radiomics have also been used in the evaluation of response to neoadjuvant treatment while still in the pre-operative phase. Delta radiomics refer to the quantitative change in radiomic feature data over two or more time points. This can track and measure changes in features over a time period, disease progression, before or after a therapy, or to monitor a continued response to treatment. In this case, it was applied to measure the changes between the pre-neoadjuvant CT images and the pre-operative imaging. Han et al. used a delta radiomics model to predict response to neoadjuvant chemoimmunotherapy in 206 patients with clinical stage IIA-IIIB NSCLC. They found that the delta radiomics model outperformed the pre-treatment radiomics model in the two external validation sets (AUC = 0.83, 0.72 vs. 0.48, 0.61). Immune-related RECIST (iRECIST) response was also evaluated, and a combined delta radiomic-iRECIST model had AUCs of 0.85 and 0.67 compared to 0.65 and 0.47 for solely iRECIST in the external sets (148). Similarly using delta radiomic features, Xiong et al. and Bao et al. built models for predicting pCR and MPR(validation AUCs = 0.72 and 0.75, respectively) (149, 150).

Lastly, radiomics has also been used to study the decision of the extent of the tumor resection, specifically sub-lobar versus lobar resection for early-stage tumors. From 248 modified covariates which included both clinical and CT-radiomic features in training and test sets of 369 clinical stage IA pure-solid NSCLC, Yan et al. identified and used four clinical features and ten radiomic features to develop a predicted score for each patient (stratified as positive-score and negative-score). In the positive-score group, sub-lobar resection was significantly associated with worse OS (hazard ratio [HR] 2.56, 95% CI 1.35-4.85, p < 0.001) and trended towards worse RFS (HR 2.13, 95% CI 0.87-5.24, p = 0.09). However, for the negative score-group, better RFS was found with a sub-lobar resection (HR 0.47, 95% CI 0.26-0.85, p = 0.01) (151). Aiming to investigate the same question, Tan et al. utilized a cohort of 657 patients with resection for lung adenocarcinoma ≤ 2cm among whom 345 (52.5%) underwent a lobectomy and 312 (47.5%) underwent a sub-lobar resection. Using pre- and post-operative high-resolution CT imaging, they identified four CT features: both spiculation and air bronchogram, spiculation only, air bronchogram only, and neither sign present. Then, multiplanar volumetric reconstruction was used to measure the maximal diameter of the solid component. Although there was no significance in terms of 5-year RFS or OS between the two procedures, they did find differences with imaging features. Those with solid components > 5.6 mm in size had significantly worse RFS and OS (p = 0.049 and 0.018 respectively) along with those with spiculation, though a positive air bronchogram was protective. On a subgroup analysis comparing survival with lobar vs. sub-lobar resection within four different populations—those with both spiculation and air bronchogram, with spiculation but no bronchogram, with bronchogram but no spiculation, or neither—those with spiculation but no air bronchogram who underwent lobar resection instead of sub-lobar had improved OS (81.5% vs. 71.5%, p = 0.026) (152). Although these studies are far from definitive, they do indicate that radiomic modeling has the potential to suggest certain patient populations might benefit from different resections. A summary table of the above studies that use radiomics-based models to predict tumor responsiveness to treatment is provided in **Table 5**.

**Table 5:** Key studies using radiomics to predict response to therapy.

Authors	Year	Study type	Inclusion criteria	Study size	Key findings^a^
Liu et al. (144)	2025	Retrospective single-center cohort	Patients with resectable stage IB-IIIB NSCLC who received neoadjuvant chemoimmunotherapy	Training (n = 147) Validation (n = 63)	PyRadiomics extracted PET/CT features in a combined radiomic-clinical model can predict complete pathologic response to neoadjuvant chemoimmunotherapy (AUC = 0.82)
Fan et al. (145)	2025	Retrospective single-center cohort	Histologically confirmed stage II-III NSCLC with pre-treatment CT scans prior to neoadjuvant chemoimmunotherapy	Training (n = 151) Validation (n = 65)	A CT radiomic feature based model using both tumoral and peri-tumoral features could predict pathologic complete response to chemoimmunotherapy (AUC = 0.75)
Wang et al. (147)	2024	Retrospective single-center cohort	Resectable NSCLC without EGFR or ALK mutation who received neoadjuvant chemoimmunotherapy	Training (n = 148) Validation (n = 63)	Combined clinical-radiomic model using PyRadiomics extracted features could predict major pathologic response to neoadjuvant chemoimmunotherapy
Han et al. (148)	2024	Retrospective multicenter cohort	cStage IIA-IIIB NSCLC patients who received neoadjuvant chemoimmunotherapy and surgery	Training (n = 114) Validation (n = 50)	A delta radiomics based model outperformed the pretreatment radiomics and clinical radiographic models (AUC = 0.83)
Yan et al. (151)	2025	Retrospective single-center cohort	Pure-solid cStage IA NSCLC undergoing resection	Training (n = 369) Validation (n = 369)	A risk score was developed with a clinical-radiomic model. Positive score was associated with worse OS with sublobar resection, and negative score was associated with better RFS with sublobar
Tan et al. (152)	2025	Retrospective single-center cohort	Patients with resected ADC ≤ 2 cm	N = 657	Improved OS in patients with lobar resection vs. sublobar who have nodule spiculation but no bronchogram

NSCLC, non-small cell lung cancer; AUC, area under the curve; OS, overall survival; RFS, recurrence-free survival; ADC, lung adenocarcinoma.

^a^ AUC values provided are all from validation sets when able.

## 3 Discussion

Overall, there is an exciting wealth of venues to which radiomics can contribute positively to the accurate prognostication of lung cancer patients. As patients increasingly receive personalized treatment plans that go beyond the traditional staging classification, radiomics features that can be obtained non-invasively and accurately can further guide towards the best management plan for distinct patient cases.

Several radiomic tools have shown great promise for aiding in the treatment of NSCLC. Both internally developed modeling algorithms and more freely available software have shown predictive capability and clinical applicability. Now that large-scale clinical trials have shown oncologic adequacy with sublobar resections beyond the previous standard lobectomy, and neoadjuvant chemoimmunotherapy has become approved for resectable tumors beyond stage IA, two questions follow: Which patients are the ideal patients for these treatments? And who might be better served with a lobectomy vs. sublobar resection? The ability of radiomic models to identify histologic subtypes and pathological features can be quite useful. Biopsy modalities can provide variable amounts of tissue with inconsistent diagnostic yield, and fine-needle aspirates fail to provide information about the underlying tissue morphologic architecture (153–155). The clinician may know that a lesion is malignant but have no further information about the relative degree of aggressiveness. Furthermore, tumors are heterogeneous. The World Health Organization (WHO) classifies a tumor as high-grade with > 20% of a high-grade histological subtype (solid or micropapillary) (156). If the biopsy instrument samples from a part of a tumor without that fractional amount of high-grade pattern, then a false impression on the tumor aggressiveness might be given. The ability for radiomic software to extract features and feature patterns that may in turn be associated with certain histological subtypes can help parse through tumor heterogeneity and pick up fractional portions of high- vs. low-grade patterns, which would be vital.

Knowing the relevant genomic mutational burden and proteomic expression with the increasingly widespread use of perioperative ICI is also important. For resectable stage IB-IIIA NSCLC, neoadjuvant chemoimmunotherapy confers a survival benefit, but those with *EGFR* mutations or *ALK* gene fusions are not eligible for nivolumab or durvalumab (17–19, 83). In resource-rich settings, sending out for a gene panel and pathology lab staining for PD-L1 and PD-1 are part of the standard of care, but this may not be immediately accessible everywhere. A reliable predictor for common key genetic mutations and proteomics could be hugely important in resource-limited settings.

CANARY is a tool that has shown itself to be quite useful and applicable for assessing relative risk in known lung ADC. Its strengths are its ease of use with the semi-automated seed growing for mask segmentation on CT image slides and automatic processing and SILA generation. All the user is required to do is the selection of the nodule and then adjust borders after the seed is grown. Highlighting its ease of use is a low inter-observer variability and high reliability with nodule segmentation across users of varying levels of clinical experience, spanning an undergraduate student to a resident to a thoracic radiology fellow (76, 86). The generated SILA has been reliably associated with both patient recurrence and treatment paradigm-changing findings such as OLNM, highlighting its real-world capability (78, 79, 87). Its drawbacks at this time are that it is validated only with ADC, not SCC, and it requires a diagnosis of cancer to be applicable. Other models have been developed for predicting invasive malignancy from undifferentiated nodules.

PyRadiomics is also a useful tool for development of radiomic models. It is freely available with accessible user guides and documentation of its underlying analysis. Its widespread use amongst research groups developing radiomic predictive models underline its usefulness in extracting key radiomic features. It functions differently than CANARY though. It extracts the radiomic data in order to build a predictive model, but it does not have a predictive modeling software built in like CANARY. However, the popular usage of the program does allow for crossover between individually developed models as there will be common radiomic features will have been extracted the same way.

Aiding in the accurate staging of NSCLC is one of the main goals of these pre-treatment predictive radiomic models. Increased detection of lymph node metastasis would be of high value in the preoperative setting because this would upstage the patient and make them eligible for neoadjuvant treatment. The post-operative pathologic upstaging that occurs with the discovery of OLNM means that the patient was denied pre-operative treatment that may have benefited them. A determination of a high risk for OLNM is also valuable because it can help the surgeon decide if a more extensive resection might benefit the patient. Current guidelines dictate the sampling of one N1 lymph node station and three N2 lymph node stations during a pulmonary oncologic resection, for either a lobar or sublobar resection (83, 157, 158). However, a sublobar resection will miss many of the N1 lymph nodes that will otherwise have been harvested in a lobectomy. If there is a high risk of OLNM, a surgeon may elect for a lobectomy, even if the patient otherwise would meet other criteria for sublobar resection, to ensure an intraparenchymal lymph node harboring unknown metastatic cells is not missed.

One of the major strengths of this review and the current literature is that there is currently a wealth of studies being published on the topic. AI has become an extremely popular subject for exploration, and we would anticipate that the number of studies and models will only continue to grow. The vast majority of the studies included in this review are from within the past three years. As AI continues to develop and become integrated into the practice of healthcare, it is likely that it will be used to interpret radiomic data independently. Another major strength is that the included studies illustrate that radiomic modeling can be integrated into clinical practice at every step of the pre-operative stage. It can be used to predict tumor genomics and mutant protein expression for personalized drug targeting, lymph node metastasis for operative planning, and then prognosis and response to treatment. Furthermore, this study just focused on resectable NSCLC, but many studies have similarly been published on responses to treatment for non-resectable and metastatic disease.

Radiomics based studies themselves have several limitations and can be prone to bias in the data. Image acquisition factors such as voxel size or scanner protocols can impact the reproducibility of radiomic features across multiple patients. Within the same subject, repeatability of features is not always consistent (159–162). One study using PyRadiomics extraction of 1080 features found that 22.5% of features had good reproducibility and 46.1% had good repeatability (162). Harmonization and data normalization can help standardize data across different acquisitions, but it can be imperfect (160). Even the image pre-processing for feature extraction can introduce bias to the downstream data. Voxels may be resampled prior to feature extraction to adjust voxel size or to select for gray level (163, 164). Even for feature extraction and selection, there are multiple different extraction software packages available, and not all extract the same feature library (165).

Another important limitation in radiomics studies is the potential for “overfitting” of the model. With the high number of radiomic features that can be extracted, particular feature patterns can be detected and tightly associated with the outcome of interest within the training image set. Therefore, a validation or external test set of images is vital in order to show generalizability of a predictive model based on a detected radiomic feature signature. Furthermore, because many of these studies are single-institution based, the image sample sizes can be limited which can lead to a higher likelihood of overfitting, or they may be poor representative sample of the general population. While radiomic feature based predictive models have great potential for integration into clinical practice, understanding the methodology for how a particular model was developed is crucial to deciding whether it might be applicable to one’s own clinical practice.

One of the significant limitations of this review in particular is that none of the studies are randomized prospective trials and are instead all retrospective database studies with models retrospectively fit to existing cohorts. Therefore, there is a strong potential for bias in reporting positive results for the usage of these radiomic studies. To truly show its benefit, one of the included predictive models would need to be used prospectively to help make decisions with resulting patient outcomes analyzed. Although we tried to maintain minimum-size external validation sets with consistent patient population characteristics, most confounding variables for the individual patient populations within each study could not be controlled. This is made more prominent in the setting of the multitude of statistical analyses covered, imaging acquisition processes, and the variety of outcomes of interest covered. This makes it very hard to directly compare individual studies. Another significant limitation of this particular review is that none of the predictive models are primed yet for seamless integration into clinical practice. Although most of the models provide easy-to-use nomograms, they are created on limited data sets, typically in East Asian populations from one to three hospitals, and they have yet to be tested against large populations. Furthermore, image segmentation and analysis can take a while, and a more streamlined program would be needed for integration into a busy daily practice. Next steps in this field will be the validation of models against larger and diverse patient populations and the refinement of programs to make them usable clinically.

## 4 Conclusions

Advancements in the management of early-stage NSCLC have opened up new treatment methodologies with the advent of immunotherapy and sublobar resections. A deeper understanding of tumor biology is required to be able to select appropriate patients for different treatments. Radiomic predictive models can offer non-invasive insights into the underlying tumor genomics, protein expression, histopathological makeup, lymphatic spread, and eventual outcomes. Several tools have already been developed to use radiomics to evaluate risk specifically in NSCLC. The integration of these models into clinical practice will aid clinician decision making and offer more personalized treatment for patients.
